# Improving motor and cognitive recovery following severe traumatic brain injury using advanced emotional audio-video stimulation: Lessons from a case report

**DOI:** 10.1097/MD.0000000000026685

**Published:** 2021-08-06

**Authors:** Rosaria De Luca, Patrizia Pollicino, Carmela Rifici, Cristina de Cola, Luana Billeri, Silvia Marino, Simona Trifirò, Elisabeth Fiumara, Maria Randazzo, Placido Bramanti, Michele Torrisi

**Affiliations:** IRCCS Centro, Neurolesi Bonino Pulejo, Messina, Italy.

**Keywords:** minimally conscious state (MCS), Neurowave emotional stimulation (NES), standard sensorial stimulation, traumatic brain injury (TBI)

## Abstract

**Rationale:**

It is estimated that about 6 million people suffer from severe traumatic brain injury (TBI) each year (73 cases per 100,000 people). TBI may affect emotional, sensory-motor, cognitive, and psychological functions with a consequent worsening of both patient and his/her caregiver's quality of life. In recent years, technological innovations allowed the development of new, advanced sensory stimulation systems, such as Neurowave, to further stimulate residual cognitive abilities and, at the same time, evaluate residual cognition.

**Patient concern:**

An 69-year-old Italian man entered our neurorehabilitation unit with a diagnosis of minimally conscious state following severe TBI. He breathed spontaneously via tracheostomy and was fed via percutaneous gastrostomy. At the neurological examination, the patient showed severe tetraparesis as he showed fluctuating alertness and responsiveness to external stimuli and opened the eyes without stimulation.

**Diagnosis:**

Patient was affected by subarachnoid hemorrhage and frontotemporal bilateral hematoma, which were surgically treated with decompressive craniotomy and subsequent cranioplasty about 6 months before.

**Interventions:**

The patient underwent a neuropsychological and clinical evaluation before (T0) and after a conventional rehabilitation cycle (T1), and after a Neurowave emotional stimulation-supported rehabilitative cycle (T2).

**Outcomes:**

Following conventional rehabilitation (T1), the patient achieved a partial improvement in behavioral responsiveness; there was also a mild improvement in the caregiver's distress. Conversely, Neurowave emotional stimulation session determined (at T2) a significant improvement of the patient's behavioral responsiveness, cognition, and in the caregiver's distress. The P300 recording in response to the NES showed a significant change of P300 magnitude and latency.

**Discussion:**

Our data suggest that emotional-integrated sensory stimulation using adequate visual stimuli represents a beneficial, complementary rehabilitative treatment for patients in minimally conscious state following a severe TBI. This may occur because stimuli with emotional salience can provide a reliable motivational resource to stimulate motor and cognitive recovery following severe TBI.

## 1 Introduction

Traumatic brain injury (TBI) is a non-degenerative, non-congenital insult to the brain from an external mechanical force, possibly leading to permanent or temporary impairment of cognitive, physical, and psychosocial functions, with an associated diminished or altered state of consciousness.^[1,2]^

TBI prevalence is 12% to 17% in males and 8.5% in females.^[3]^ In particular, it is estimated that about 6 million people suffer from severe TBI each year (73 cases per 100,000 people).^[4]^ Among the survivors of moderate to severe head injury, 31.8% of patients die or need hospitalization in a specialized health center; 44% are unable to return to work, and 88% of the patients with mild TBI have white matter damage, with negative repercussions on functional outcomes.^[5]^ Moreover, TBI may affect emotional, sensory-motor, cognitive, and psychological functions with a consequent worsening of both patient and his/her caregiver's quality of life.^[6,7]^ Given that TBI is the most common cause of long-term disability and death among young adults, it represents an enormous socio-economic and health care burden.^[3]^

Therefore, the role of rehabilitation for patients with TBI sequelae, including the chronic disorders of consciousness, such as the minimally conscious state (MCS) and the unresponsive wakefulness syndrome, is of outstanding importance. Conventional rehabilitation (CR) includes the prevention of deterioration and the maintenance of the sensory-motor system and other vital body functions to avoid medical complications and maximize functional outcomes. Among CR approaches, sensory stimulation (SS) consists of the application of environmental stimuli by an external agent for promoting arousal and behavioral responsiveness.^[8]^ SS programs usually consist of presenting a repetitive, frequent, and moderate-to-high intensity simple stimulation,^[9]^ and they vary in intensity and frequency of intervention, as well as the targeted senses. At a minimum, most programs included stimulation of visual, auditory, olfactory, kinesthetic, and tactile senses.^[10,11]^ Particularly, the processing of emotional information is prioritized in the cognitive system^[12]^ and the sensory processing is enhanced by emotion.^[13]^ Therefore, emotional stimuli may receive privileged access to attention and awareness systems.^[14]^ In this regard, stimuli with autobiographical relevance can represent a valuable option for SS. The retrieval of autobiographical memories involves multiple processes, that is, episodic memory, personal semantic knowledge, visual imagery, emotional processing, self-referential, and control executive processes,^[15,16]^ and accordingly engages a large network of brain regions (predominantly left-lateralized and medial brain regions).^[17]^

In recent years, technological innovations allowed the development of new, advanced SS systems, such as PC-based rehabilitative programs or virtual reality training, to further stimulate residual cognitive abilities and, at the same time, evaluate residual cognition.^[18]^ Such approaches have been proven effective in recovering cognitive and sensory-motor functions in neurological patients, including TBI.^[19,20]^ Among the available systems, the Neurowave (Khymeia Group; NoventaPadovana; Italy) is an innovative integrated system for the administration of multi-sensory stimulation and the simultaneous acquisition and analysis of bio-physiologic parameters in neurological patients with limited cognitive abilities.

The present study was aimed at preliminarily assessing the usefulness of SS, using the Neurowave system, in stimulating the behavioral responsiveness, cognitive and motor processing in subjects in MCS resulting from a severe TBI.

## 2 Case description

An otherwise healthy 69-year-old Italian man entered our neurorehabilitation unit with a diagnosis of MCS following severe TBI (car accident) characterized by subarachnoid hemorrhage and frontotemporal bilateral hematoma, which were surgically treated with decompressive craniotomy and subsequent cranioplasty about 6 months before. Family history was unremarkable. He breathed spontaneously via tracheostomy and was fed via percutaneous gastrostomy. At the neurological examination, the patient showed severe tetraparesis and he was in an MCS, as he showed fluctuating alertness and responsiveness to external stimuli (ie, he reproducibly attempted to move the hands, attempted to localize noxious stimulation, followed a target with the eyes, and made some oral movements) and opened the eyes without stimulation. Therefore, his communication capacity was labeled as non-functional but intentional. Laboratory tests were within the normal range. The patient also underwent a psychometric and clinical evaluation (T0), consisting of the levels of cognitive functioning (LCF), the Coma Recovery Scale-Revised (CRS-R), and the Extended Glasgow Outcome Scale (GOSE). Furthermore, the Global Severity Index/Symptom Checklist-90-R (SCL-90-R) was administered to the patient's caregiver (his wife) as ad hoc behavioral scales.

The LCF is one of the earlier developed scales used to assess cognitive functioning in post-coma patients.^[21]^ The CRS-R is used to assess patients with a disorder of consciousness, including coma, unresponsive wakefulness syndrome, and MCS.^[22,23]^ The CRS-R consists of 23 items, grouped into 6 sub-scales (auditory, visual, motor, oromotor, communication, and arousal). The lowest score on each sub-scale represents reflexive activity and the highest represents behaviors mediated by cognitive input. The total score ranges between 0 (worst) and 23 (best). In addition, we administered the Disorders of Consciousness Scale (DOCS), which is a bedside test measuring neurobehavioral functioning during coma recovery by describing the levels of neurobehavioral integrity and responsiveness to test stimuli.^[24]^ We also adopted this behavioral scale as both the minimal detectable change (MDC95) and the minimally clinically important difference (MCID) have been defined. Further, we estimated the GOSE,^[25]^ which is a global scale for the functional outcome that provides a detailed categorization into 8 categories by subdividing the categories of severe disability, moderate disability, and good recovery into lower and upper categories. Lastly, we administered the SCL-90-R,^[26]^ which is a multidimensional self-report measure, assessing the severity of current psychological symptoms and distress (including somatization, obsessive-compulsive, interpersonal sensitivity, depression, anxiety, hostility, phobic anxiety, paranoid ideation, and psychoticism). Furthermore, the number of symptoms endorsed and intensity of distress, the average level of distress for those items that were endorsed, and the total symptoms endorsed/breadth of distress are enclosed as global indices of psychological distress during the past 7 days. It consists of 90 items, each rated from 0 (Not at All) to 4 (Extremely).

The patient was thus provided with an intensive CR program for 2 months, including cognitive and motor conventional training based on a face-to-face approach between the therapist and the patient in a protected contest.

**Figure 1:**
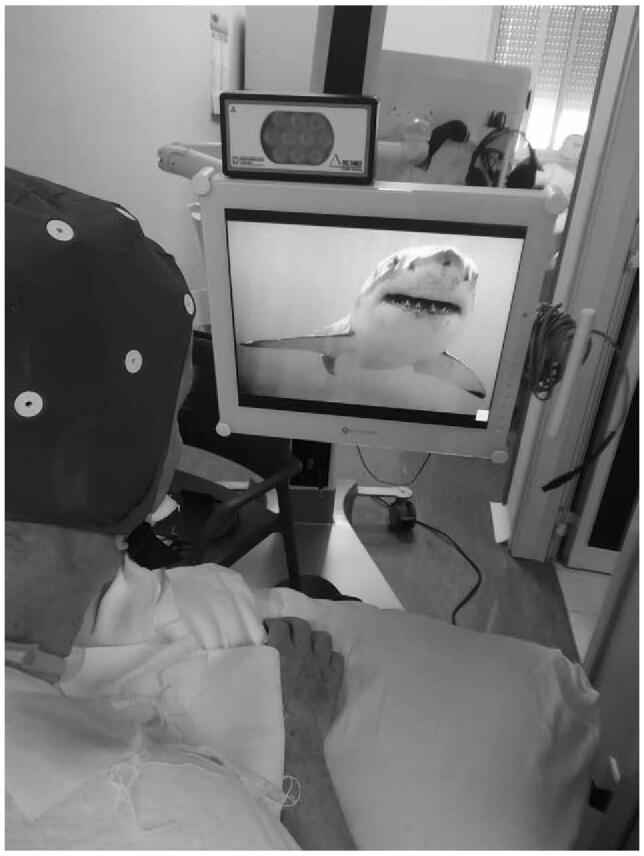
Patient during a NES session. NES = Neurowave emotional stimulation.

CN is the usual standard treatment of movement disorders caused by impairments of joints and the muscles, implemented in current clinical practice after brain injury. In practice, the physiotherapist has applied specific mobilization-based treatment techniques such as manipulative therapy and standard sensory audio-video stimulation, carefully to limb and/or core muscle strengthening exercises. Each traditional rehabilitative session has been made a quiet place away from any form of source of distraction, face to face with the therapist, for a duration of 60 minutes. Mobilization, strengthening, and stretching constitute the 3 main treatment approaches in our standard physiotherapy program used (Fig. 1). In particular, the therapist performed passive mobilization to the 4 limbs, with a focus on joint ROMs with the patient positioned in supine beds with head and trunk aligned; moreover, the therapist has used motor tasks including stretching of muscles (sternocleidomastoid, trapeze, large, and small pectoral) with specific exercises of reduction of the stiffness, the cervical tract and rotation enhancement, task-oriented to tilt and flex-extension of the head. On the bedside and feet resting on the ground, the patient is stimulated through two-way (personal and peripersonal left-center-right side) verbal stimuli, sound (using a bell; personal music), and visual (using photos familiar o personal tools such as emotionally significant objects) with gradual passive and active exercises of approach and removal of the trunk, sitting, with a cube behind the back.

The patient was reassessed after the end of the CR (T1). As he showed only a mild improvement limited to LCF (Table 1), we implemented the use of NES within the CR program after 15 days of rest. In this regard, we adopted an intensive and repetitive task-oriented sensory-motor stimulation, using an emotional therapy with audio-video personalized materials. Each NES session was implemented in the patient's current clinical practice, with a duration of 60 minutes, for 6 sessions weeks for a total of 24 weeks see (Fig. 1). In addition, the patient underwent P300 recording during the first and last 3 NES sessions consistently.^[27]^

**Table 1:** Clinical and psychometric scores at baseline (T0), post-CN/pre-NES (T1), and post-NES (T2).

		Domains	T0	T1	RCI value	T2	RCI value
Patient's clinical scale	LCF range 0–8	Cognitive functioning	2	2	0	3	−0.57
	CRS-R range 0–23	Behavioral awareness	11	13	−0.55	17	−1.7
	DOCS range 0–46	Behavioral awareness	20	17	−1.7	28	−2.1
	GOSE range 1–8	outcome	3	3	0	4	−0.5
Caregiver's psychophysical scale	SCL-90-R range 0–360	Caregiver's distress	270	216	3.67	151	5.28

Neurowave is an innovative and technologically advanced device that allows the programming and automated administration of sensory stimuli (such as images, movies, and sounds, including patient-specific recordings) and the simultaneous monitoring of multiple bio-physiological signals. Neurowave is an advanced system able to monitor event-related potentials induced by neurosensory stimulation. Neurowave is an innovative integrated system for the administration of multi-sensory stimulation and the simultaneous acquisition and analysis of bio-physiologic parameters of patients in a vegetative and minimally conscious state. It is an innovative technology “open” platform, extremely flexible, targeted to clinical research applications in the field of serious disorders of consciousness during all stages of the patient status. Neurowave includes an advanced multi-sensory stimulation system and a simultaneous and synchronized system of acquisition of bio-physiologic signals, accompanied by a comprehensive assessment system of the eventual correlations between sensory stimulation and changes in the patient's status. Neurowave has excellent construction and ergonomics features by putting together all the functionalities of the system in a single chassis that is placed at the patient bedside, though requiring minimum space even in sensitive environments such as intensive care rooms. The system can be easily moved from 1 bed to another.

After the last NES (T2), the patient was provided with the same neuropsychometric battery at baseline and electroencephalogram recording in resting state.

Because of the patient's disturbance of consciousness, we asked for the daughter's consent to publication.

## 3 Results

At the baseline assessment (T0), the patient showed a global impairment in cognitive and motor function (LCF and GOSE) and in behavioral responsiveness (DOCS and CRS-R). This clinical picture was paralleled by high-level distress in the patient's caregiver (SCL-90-R). The patient was provided with intensive CR, after which (T1) he achieved an improvement in behavioral responsiveness (DOCS) that was superior to MDC95 (6 points) but not to MCID (9 points) (Table 1). This treatment also provided the caregiver with significant distress relief (SCL-90-R). On the contrary, the provision of the patient with NES session determined (at T2) an improvement in both behavioral responsiveness scales (CRS-R and DOCS); in particular, the increase in DOCS scoring achieved the MCID also. Furthermore, there was a further, high improvement of caregiver's distress (Table 1). Additionally, ENS device furnished us with P300 recording in response to the appropriate visual stimuli. We found that the latency of N100 was not significantly affected, whereas that of N200 and P300 showed a significant decrease (both RCI = 10). The amplitude of N100 and N200 significantly increased (RCI = −3.7 and RCI = −6.6, respectively), whereas that of P300 did not significantly change.

## 4 Discussion

The purpose of the study was to evaluate the effectiveness of an advanced SS approach for improving motor and cognitive functions in a subject in MCS following severe TBI. To the best of our knowledge, this is the first study to investigate the effect of this rehabilitative intervention using the Neurowave System. Multisensory environment is found in ecological settings, which have been well recognized for serving as a resource for recovery and rehabilitation.^[28,29]^ TBI-induced brain damage produces sensory deprivation.^[30]^ Thus, the rationale for treatment is to enrich the environment and promote neural plasticity to prevent any additional detrimental effects on an already damaged brain.^[10]^ Multisensory environments are found in nature, recognized as beneficial to many medical conditions. Interventional approaches resort to variations of multisensory stimulation such as multimodal stimulation of the senses,^[31]^ music therapy,^[32]^ or verticalization protocol using a tilted table with an integrated stepping device.^[33,34,35]^ Particularly, multisensory stimulation is effective in enhancing the recovery process of severely brain-injured patients with disorders of consciousness.^[36]^ Particularly, emotional stimuli capturing attention are prioritized in the cognitive system and intensify sensory integration.^[13,27,14]^ Emotional and biographical stimuli were directly provided through NES, a significant effect was found when addressing the patient with her or his own name by their caregiver with audio-video and materials. Further research must be conducted to reach more concrete conclusions regarding the benefits of emotional salience on increasing volitional behaviors in cases of severe TBI.

Overall, the results pointed out limited effectiveness of CR on cognitive functioning, behavioral responsiveness, and caregiver burden; instead, NES determined a significant improvement in behavioral responsiveness and in caregiver's distress. These findings are consistent with the improvements in arousal degree, interaction with the environment, psychomotor initiative, and the level of consciousness as reported in the literature.^[33]^ In keeping with these issues, we can speculate that promoting motivational stimulation and increasing sensory inputs using multisensory stimulation may modulate the neural activity of different sensorimotor networks, resulting in a better generation of motor programs. This interpretation is supported by the fact that P300 magnitude significantly increased, which indicates a potentiation of cognitive processes related to sensory and emotional stimuli, which in turn brought to an enhancement of motor programs. Actually, observing an emotional image entrains the motor programs related to the emotional content, consistently with embodied cognition theories.^[37]^ Even though this issue is neither directly tested nor demonstrated in our study, we found an improvement in motor outcome, which may support our hypothesis. This may also explain why NES provided the patient with better outcomes as compared to CR, which could be considered as less motivating and cognitively entraining owing to its lower sensory stimulation activity.

In our study, we observed that the use of NES was more effective than CR also regarding better management of caregiver's distress syndrome. This is an important issue, as there is a definitive existence of psychological problems also in overburdened caregivers. In particular, the time since injury had a considerable impact on the family homeostasis. The strongest stress allaying factor was proved to be a therapeutic intervention in the form of problem-solving training.^[38]^ We hypothesized that caregiver's distress reduction resulted from the improvement of motor and cognitive functions as observed by family members, where the emotions play a decisive role. In the current literature is evident that stimuli with emotional salience also provide a reliable motivational resource.

## 5 Limitations and conclusions

An important limitation of our study concerns the short follow-up; for this reason, it will be essential to extend the target population to confirm this promising result in the next studies. Furthermore, it is not known whether an advanced system such as Neurowave affects the intervention of the therapists in terms of their mood improvement or increased motivation to stimulate cognition's patient, which may have influenced the response to the intervention. Further studies may use additional measurements to evaluate more specifically motivational aspects such as participation performances (by patients and by therapists) or reactions to specific emotionally salient stimuli. In addition, physiological measurements such as salivary cortisol and vital parameters such as to evaluate the effect of natural settings on the stress level of patients with severe brain injuries would be interesting.

Notwithstanding, our findings suggest that SS with advanced systems may provide patients with TBI motor and cognitive sequelae with complementary beneficial rehabilitative treatment. The sensory approach may improve adaptive, goal-oriented behaviors hence helping to restore functional interactive communication. Stress reduction was also found to be responsive to the type of intervention given to their families according to the specific challenges faced by the caregiver and would help them deal better with their newfound role. Despite further and larger studies that should be fostered to confirm these promising findings, our data advocate, in a more global perspective, for the restorative benefits of advanced technologies as a relevant complimentary resource for public health.

## Author contributions

**Conceptualization:** Rosaria De Luca.

**Data curation:** Cristina De Cola, Luana Billeri.

**Formal analysis:** Cristina De Cola.

**Investigation:** Luana Billeri, Simona Trifirò, Elisabeth Fiumara, Maria Randazzo.

**Methodology:** Cristina De Cola.

**Project administration:** Patrizia Pollicino, Carmela Rifici, Silvia Marino.

**Supervision:** Silvia Marino, Placido Bramanti.

**Visualization:** Michele Torrisi.

**Writing – original draft:** Rosaria De Luca, Michele Torrisi.
